# Night shift-induced circadian disruption: links to initiation of non-alcoholic fatty liver disease/non-alcoholic steatohepatitis and risk of hepatic cancer

**DOI:** 10.20517/2394-5079.2024.88

**Published:** 2024-10-30

**Authors:** Anjali Singh, Baby Anjum, Qulsoom Naz, Sana Raza, Rohit A. Sinha, Mohammad Kaleem Ahmad, Abbas Ali Mehdi, Narsingh Verma

**Affiliations:** 1 Department of Physiology, King George’s Medical University, Lucknow 226003, India; 2 Department of Neurology, Sanjay Gandhi Postgraduate Institute of Medical Sciences, Lucknow 226014, India; 3 Department of Medicine, King George’s Medical University, Lucknow 226003, India; 4 Department of Endocrinology, Sanjay Gandhi Postgraduate Institute of Medical Sciences, Lucknow 226014, India; 5 Department of Biochemistry, King George’s Medical University, Lucknow 226003, India; 6 Era University, Lucknow 226003, India; 7 Hind Institute of Medical Sciences, Sitapur 261304, India

**Keywords:** Night shift, circadian disruption, liver homeostasis, non-alcoholic fatty liver disease (NAFLD), hepatic cancer

## Abstract

The circadian system plays a crucial role in regulating metabolic homeostasis at both systemic and tissue levels by synchronizing the central and peripheral clocks with exogenous time cues, known as zeitgebers (such as the light/dark cycle). Our body’s behavioral rhythms, including sleep-wake cycles and feeding-fasting patterns, align with these extrinsic time cues. The body cannot effectively rest and repair itself when circadian rhythms are frequently disrupted. In many shift workers, the internal rhythms fail to fully synchronize with the end and start times of their shifts. Additionally, exposure to artificial light at night (LAN), irregular eating patterns, and sleep deprivation contribute to circadian disruption and misalignment. Shift work and jet lag disrupt the normal circadian rhythm of liver activity, resulting in a condition known as “circadian disruption”. This disturbance adversely affects the metabolism and homeostasis of the liver, contributing to excessive fat accumulation and abnormal liver function. Additionally, extended working hours, such as prolonged night shifts, may worsen the progression of non-alcoholic fatty liver disease (NAFLD) toward non-alcoholic steatohepatitis (NASH) and increase disease severity. Studies have demonstrated a positive correlation between night shift work (NSW) and elevated liver enzymes, indicative of hepatic metabolic dysfunction, potentially increasing the risk of hepatocellular carcinoma (HCC) related to NAFLD. This review consolidates research findings on circadian disruption caused by NSW, late chronotype, jet lag, and social jet lag, drawing insights from studies involving both humans and animal models that investigate the effects of these factors on circadian rhythms in liver metabolism.

## Introduction To The Circadian System

Circadian rhythms govern various physiological functions through intrinsic autonomous oscillators known as free-running rhythms, allowing organisms to adapt their behaviors to a roughly 24 h cycle in response to environmental changes aligned with the Earth’s day-night cycle^[1]^. These internal (endogenous) physiological rhythms are orchestrated by a master pacemaker in the hypothalamus known as the suprachiasmatic nucleus (SCN). These intrinsic “time-keeping” oscillators synchronize with the external environmental time cues known as zeitgebers, with light being the most potent zeitgeber^[2–4]^. However, improper exposure to zeitgebers, a prevalent issue in modern society, can disrupt circadian equilibrium and adversely affect human health. The central SCN clock is synchronized with peripheral clocks present in nearly every cell across various organs^[1]^.

## Central/Master Clock (Suprachiasmatic Nuclei) and Peripheral Clocks

The central clock, the SCN, in mammals uses direct neural projections via the retinohypothalamic tract (RHT) to respond to light signals received from the retina. SCN acts as a master clock that coordinates the synchronization of the peripheral tissues through autonomic and endocrine signals^[5]^. At the molecular level, the circadian clock functions through a transcription-translation feedback loop (TTFL), with about 24 h periodicity^[6]^. The SCN and peripheral clocks employ similar molecular mechanisms based on an auto-regulatory TTFL comprising positive (activating) and negative (inhibitory) feedback loops^[7]^. The core of the circadian system features crucial clock genes, including circadian locomotor output cycles kaput (CLOCK) and brain and muscle Arnt-like protein-1 (BMAL1)^[8]^. On the contrary, the negative feedback loops involve Period (PER) genes PER1/2/3 and Cryptochrome (CRY) genes CRY1/2^[9,10]^. Additionally, the Rev-Erb alpha and retinoid-related orphan receptor alpha (RORα) nuclear receptors (NRs) regulate BMAL1 transcription through competitive binding to the ROR-response element (RORE)^[11,12]^. The SCN regulates peripheral tissue clocks via multiple mechanisms, including: (1) innervation of peripheral tissues by the autonomic nervous system; (2) signaling through the endocrine system; (3) temperature control; and (4) integration of behavioral signals such as feeding^[13]^. The sympathetic and parasympathetic neurons, which receive inputs from both the SCN and hypothalamus, extend their innervation to a broad spectrum of peripheral organs, encompassing the liver, muscles, adipose tissue, intestine, and heart. Peripheral clocks are further influenced by additional zeitgebers, including exercise, sleep patterns, and meal timing^[14]^. Disruption of circadian rhythms caused by improper meal timing, night shift work (NSW) schedules, or sleep deprivation often disturbs the synchronization between the body’s internal clock and the external environment. This desynchronization may affect the coordination between the central and peripheral biological processes within an organism. Such disturbances have been linked to the onset of obesity, metabolic disorders^[15]^, and several types of cancers^[16]^. Shift workers such as firefighters (exposed to carcinogenic chemicals during fires) and painters (exposed to harmful solvents and pigments) are at elevated cancer risk^[17]^. The detrimental health impacts of these disruptions include impaired glucose metabolism, alterations in endocrine and immune functions, elevated blood pressure, and heightened levels of inflammatory markers associated with shift work^[18–24]^. Night shift schedules may also disturb the 24 h rhythms of urinary metabolites among police officers on rotating shifts^[25]^.

### Peripheral circadian gene expressions in peripheral blood mononuclear cells and shift workers

There is a wealth of literature demonstrating the effects of night shifts on circadian genes (CGs). Using samples from beard follicles, a study examined clock gene expression in Japanese men across three distinct population cohorts: (1) those who work one night shift and then take the day off; (2) those who work on three or more consecutive night shifts (factory workers); and (3) those who work only daytime hours. The expression of Period 3, Nr1d1, and Nr1d2 was investigated using quantitative polymerase chain reaction (qPCR). Compared to the consecutive-night group, the patterns of Period 3 and Nr1d2 expression in the daytime and one-night groups were more alike. It suggests that, depending on the shift pattern or type, working night shifts modifies the circadian Period 3 and Nr1d2 expression rhythms and levels^[26]^. Other studies described how the night shift affects peripheral CGs and circadian-controlled genes (CCGs) linked to breast cancer. The authors measured the levels of cortisol and melatonin in plasma, as well as the levels of CCGs [erythrocyte sedimentation rate (ESR1 and ESR2)] and peripheral CGs (PER1, PER2, PER3, and BMAL1) in peripheral blood mononuclear cells (PBMCs). In day shift nurses, the 24 h rhythms of cortisol and melatonin aligned with light/dark cycles corresponding to their shifts, and these rhythms were reflected in the mRNA expression of PER2, PER3, BMAL1, and ESR2. These rhythms peaked in the morning. In contrast to day shift nurses, night shift workers displayed abnormalities in the rhythmic expressions of the PER2, PER3, BMAL1, and ESR2 genes. As a result, there was a reduced inverse connection between PER2 and BMAL1^[27]^. The study compiled fifteen epidemiological research papers, five of which focused on shift work employment, and identified BMAL1, BMAL2, CLOCK, neuronal PAS domain protein 2 (NPAS2), CRY1, CRY2, PER1, PER3, and TIMELESS as potential risk variants for breast cancer^[28]^. The study evaluated clock gene expression in two peripheral clocks, oral mucosa cells and PBMCs, in 11 police officers who worked the night shift and measured central clock markers, urinary 6-sulfatoxymelatonin, and salivary cortisol. The morning/evening difference seen at baseline in PBMCs vanished after a week of working nights, and PER1-3 and Rev-ERBα expression in oral mucosa cells lost rhythmicity. It is thought that shift work-related medical illnesses may be significantly affected by molecular circadian disruptions^[29]^. The study evaluated the resetting effects of bright light exposure on peripheral clock indicators, such as clock gene expression in PBMCs, as well as central clock markers, including plasma cortisol and melatonin, in individuals who are night owls. The phase of PER1 and BMAL1 rhythms in PBMCs was delayed by approximately 2.5-3 h (P < 0.05) in response to a night schedule; no shift was seen for the other clock genes or the central markers^[30]^. In a different study, on days off from work, PBMCs were collected for RNA extraction every 3 h, and cortisol and melatonin were measured. Differential gene expression patterns between night shift and day shift participants were found using genome-wide microarray analysis of PBMCs from a group of nurses. Furthermore, regardless of shift type, there was a significant variation in the quantity of rhythmic transcripts across the individuals. It demonstrates how shift work schedules impact PBMC gene expression and circadian alignment^[31]^. Researchers looked into how NSW and genes related to the circadian and melatonin pathways affected the risk of breast cancer in Korean women. A standardized questionnaire was used to gather data on NSW and other variables, while hospital-based case-control research was conducted to analyze twenty-two polymorphisms in eleven different genes. Upon examining the primary impacts of each single nucleotide polymorphism (SNP), variations in CLOCK rs11133373 were associated with an increased risk of breast cancer. The findings of the study support the potential involvement of various loci related to circadian rhythms, melatonin-synthesizing genes, their interactions, and gene interactions with NSW in the pathogenesis of breast cancer^[32]^.

## Circadian Misalignment: A Silent Disruptor

The circadian clock is essential for regulating vital hepatic functions and cellular processes. Contemporary lifestyles, often marked by factors such as nighttime exposure to artificial light, rotating night shift schedules, social jet lag, irregular sleep-wake cycles, and inconsistent eating patterns, frequently disrupt the natural rhythm of the circadian clock, leading to “circadian misalignment.” This phenomenon is believed to contribute to global health challenges, including obesity, non-alcoholic fatty liver disease (NAFLD), and non-alcoholic steatohepatitis (NASH)^[33]^. Circadian misalignment is also associated with various disorders, including obesity, type 2 diabetes, and metabolic syndrome (MetS)^[34–37]^. Extensive evidence from studies involving both humans and animal models indicates a clear association between circadian disruption and the incidence of hepatocellular carcinoma (HCC)^[38,39]^. Additionally, the impact of NSW on increasing the risk of NAFLD, which can potentially progress to hepatic cancer, remains uncertain^[40–42]^.

## Key Factors Responsible For Circadian Rhythm Disruption

**Figure 1:**
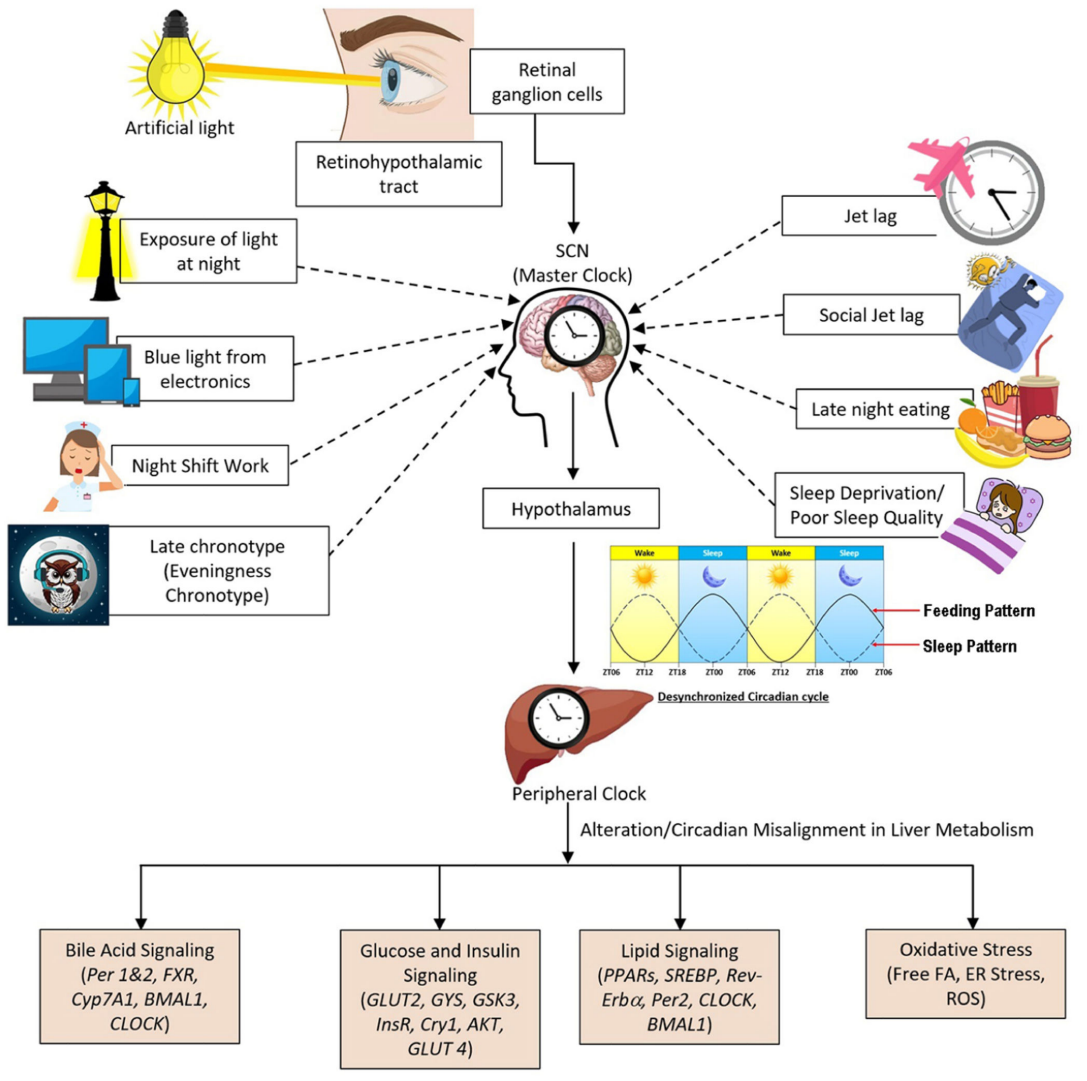
Factors responsible for circadian misalignment in liver metabolism. SCN: Suprachiasmatic nucleus; ZT: zone time; Per 1: period circadian regulator 1; Per 2: period circadian regulator 2; FXR: farnesoid X receptor; Cyp7A1: cholesterol 7 alpha-hydroxylase; BMAL1: basic helix-loop-helix ARNT Like 1; CLOCK: circadian locomotor output cycles kaput; GLUT2: glucose transporter 2; GYS: glycogen synthase; GSK3: glycogen synthase kinase-3; InsR: insulin receptor; Cry1: circadian cryptochrome; AKT: protein kinase B; GLUT 4: glucose transporter 4; PPARs: peroxisome proliferator-activated receptors; SREBP: sterol regulatory element-binding protein; Rev-Erb : NR1D (nuclear receptor subfamily 1 group D); FA: fatty acid; ER: endoplasmic reticulum; ROS: reactive oxygen species.

Artificial LAN, rotating night shifts, and social jet lag have freed modern society from the constraints of natural day-night cycles, but they have also led to behaviors that are misaligned with the body’s circadian rhythms. Circadian rhythms are significantly influenced by exposure to artificial LAN, which is particularly pronounced due to NSW. Light information from the retina of the eye is transmitted to the master circadian pacemaker, the SCN, via the monosynaptic RHT. In this pathway, light is converted into a neuronal signal by melanopsin-positive intrinsically photoreceptive retinal ganglion cells (ipRGCs) located in the retina. The neurotransmitter glutamate is released into the SCN in response to light stimulation of the retina. Glutamate plays a crucial role in regulating circadian rhythms. The SCN interacts with other brain regions, including the arcuate nucleus, paraventricular nucleus, and lateral hypothalamus, using neurotransmitters such as gamma-aminobutyric acid (GABA). The brain translates the information of light signals into an integrated response that is sent to peripheral organs through the autonomic system and hormonal signals. The peripheral clock receives the information and communicates back to the brain’s clock SCN after synchronizing with external time. The central and peripheral clocks work simultaneously to stabilize rhythms that regulate physiological functioning at the cellular and tissue levels. For example, the SCN transmits the signals of artificial light due to night shift (signals of the light-dark cycle) to the periphery of the body by directly interacting with other brain regions through the production of signaling molecules or by indirectly determining rest-activity periods that in turn regulate the feeding-fasting cycle. Disruptions in feeding and fasting rhythms due to the reverse pattern of the light-dark cycle experienced by night shift workers can lead to altered metabolic homeostasis at both the cellular and molecular levels. This misalignment can interfere with the circadian regulation of metabolic processes, ultimately impacting pathophysiology and hepatic health at a molecular or cellular level. The following are key factors responsible for circadian rhythm disruption [Figure 1], detailed below in separate sections:

### Exposure to LAN

Experimental research has demonstrated that disturbances in circadian rhythms caused by exposure to LAN may accelerate carcinogenesis in rodents^[43]^. Blue light, particularly in the 480 nm range, significantly impacts the circadian system by influencing the melanopsin-containing retinal ganglion cells (ipRGCs) that regulate the body’s internal clock^[44]^. Earlier, among newly diagnosed NAFLD adults, investigations have indicated that nighttime light exposure can reduce melatonin secretion, delay the sleep-wake cycle, disrupt circadian rhythms, and thus disrupt liver metabolism^[44,45]^. Furthermore, desynchronization between circadian clocks and the natural light-dark cycle due to LAN has been linked to health issues like sleep disorders, mood changes, metabolic issues, and even chronic conditions over time, including cardiovascular disease^[44,46]^, as well as breast and prostate cancers^[47,48]^.

### NSW

Shift work is widely acknowledged as a significant workplace hazard, impacting approximately 20% of workers in industrialized nations who participate in rotating shifts or night work outside of traditional daytime hours^[49]^. Distinct forms of shift work include “permanent NSW,” where individuals exclusively work overnight, and “rotating shift work,” which involves regular shifts that alternate between daytime and nighttime, including morning, evening, and night shifts^[50,51]^. NSW imposes considerable strain on the body’s circadian timing system. Despite its prevalence in our modern 24 h society, there is growing evidence indicating that circadian disruption associated with shift work can result in various adverse health effects^[36,52–54]^. This disruption is particularly pronounced in NSW, where Chinese steelworkers are active and eating during the typical rest phase, while sleeping and fasting during the usual active phase^[52]^.

### Late chronotype (eveningness chronotype)

In modern industrialized societies, widespread access to artificial light throughout the day and night influences individuals’ preferences in their sleep-wake cycles, known as their Chronotype^[55]^. Chronotype characterizes how an individual’s circadian rhythms vary in both behavior and biology^[56]^. Individuals are often categorized as “early” or “morning” types, referred to as Early Birds, who prefer going to bed and waking up early. Conversely, “late” or “evening” types, known as Night Owls, tend to stay up late and wake up later in the day. Like UK biobank participants, night owls are particularly susceptible to circadian disruption because their natural sleep patterns are frequently disturbed by typical work schedules compared to morning types^[57]^. The disruption of the circadian clock has been associated with the accumulation of hepatic fat and its progression to NAFLD and NASH in both animal models^[58,59]^ and, more recently, in humans^[59–61]^. A recent study indicated that individuals with a late chronotype exhibited significantly higher levels of visceral adiposity index, liver fat, and hepatic steatosis^[59]^.

### Social jetlag

Inadequate sleep is often compensated for on weekends or free days, a phenomenon known as “Social jetlag (SJL)”. This term describes the difference in mid-sleep time between workdays and free days, which reflects the degree of circadian disruption^[62]^. It is estimated that approximately 50% of workers and students experience at least 2 h of SJL, while up to 70% experience at least 1 h, due to accumulated sleep deficits during the week or adherence to fixed schedules that necessitate waking earlier than desired. Research highlights various health implications of SJL^[63]^. For example, a cohort study among the Finnish adult population identified an association between SJL and obesity, particularly among morning chronotypes^[64]^. Separate cross-sectional studies in Japan and the Netherlands, excluding shift workers, demonstrated that SJL exceeding 2 h doubled the likelihood of MetS^[65,66]^. Moreover, among prediabetic adults in Thailand, each additional hour of SJL correlated with increases in relative body fat, mean body mass index (BMI), and mean waist-to-hip ratio^[67]^.

### Late night eating

In recent decades, there has been a notable increase in eating behaviors such as skipping meals, eating later in the day, and frequent snacking among the general population^[68].^ Observational studies and small human trials indicate that these specific meal timing habits might elevate the risk of NAFLD^[69,70]^. The timing of meals and the metabolism of macronutrients are influenced by circadian rhythms. Eating at inappropriate times can disrupt these rhythms, leading to circadian misalignment. Consequently, frequent eating episodes, particularly grazing or snacking outside traditional meal times, are associated with increased susceptibility to NAFLD and obesity^[70,71]^. Modern dietary patterns frequently involve irregular and prolonged eating schedules, compounded by a Western-style diet, sedentary lifestyles, and chronic sleep deprivation. Together, these factors contribute to an increased vulnerability to metabolic disorders, including NAFLD^[72,73]^. Animal studies and small human trials suggest that confining meals to the typical active period - usually during the daytime for diurnal organisms like humans - may help reduce liver fat accumulation and improve insulin sensitivity^[58,74]^.

### Sleep deprivation/poor sleep quality

Inadequate sleep quality may contribute to an increased susceptibility to NAFLD and NASH^[75]^. Research indicates that sleep deprivation can induce hepatic steatosis and elevate alanine aminotransferase (ALT) and aspartate aminotransferase (AST) levels in mice^[76]^. Several large-scale population-based studies have shown that short sleep duration is linked to NAFLD. For instance, sleeping fewer than 5 h per night may elevate the risk of NAFLD - characterized by elevated ALT levels, a positive Fatty Liver Index, and sonographic evidence of steatosis - by 35% to 70%^[77,78]^. Moreover, evidence suggests that poor sleep quality and insufficient sleep duration among night-shift workers may increase the risk of breast cancer among women^[79,80]^.

## Shift Work Tends To Adopt Unhealthy Lifestyles

With the rise of industrialization and economic growth, shift work has become increasingly prevalent^[81]^. Individuals who work shift schedules, particularly night shifts, often experience disruptions in their sleep patterns, changes in meal timings, and exposure to artificial light during nighttime hours, which can disturb their circadian rhythms^[82]^. One possible explanation for the connection between long working hours and NAFLD is reduced physical activity during extended work periods. Excessive working hours may limit opportunities for leisure-time exercise and contribute to fat accumulation among children^[83]^. Moreover, prolonged working hours are associated with other lifestyle factors, such as smoking and alcohol consumption, among iron and steelworkers^[84]^. Additionally, NSW has been shown to exacerbate NAFLD among industrial workers^[33,41,52,85,86]^. The National Health and Nutrition Examination Survey (NHANES) reported that shift work increases the likelihood of NAFLD by 66% in lean individuals, though not in those who are obese^[40]^. Night shift working schedules positively correlate with abnormal liver function in workers without pre-existing non-alcoholic fatty liver (NAFL), indicating that circadian disruption caused by shift work schedules impacts liver health^[87]^.

## Circadian Regulation In Liver Metabolism And Homeostasis

The liver’s circadian clock is finely tuned to meal timing and is closely intertwined with energy metabolism. It plays a crucial role in orchestrating metabolic functions, prompting extensive research into its implications for diseases such as NAFLD^[61]^. Dysregulation of the circadian system disrupts the genes associated with chronic jet lag, leading to impaired hepatic metabolism and changes in circulating levels of energy, lipids, insulin, and glucose, which may contribute to hepatic cancer development^[3,39]^. Animal-based experimental models replicating conditions similar to human NSW, reveal that the dynamic nature of hepatic metabolic processes, including glucose, lipid, and cholesterol/bile acid metabolism, is influenced by feeding/fasting cycles and circadian rhythms^[88]^, and the circadian clock significantly contributes to lipid dysregulation, oxidative stress, and inflammation^[58,89]^.

Shift working schedules disrupt circadian rhythms, adversely affecting liver metabolic homeostasis, and leading to hepatic fat accumulation^[90]^. About 40% of hepatic transcriptomics exhibit circadian oscillations^[91]^, including rhythms in protein levels, posttranslational modifications, and several metabolites in mammals^[92,93]^. Recent research suggests that alterations in circadian rhythms, particularly in individuals with specific SNPs in clock-related genes like Signal transducer and activator of transcription 3 (STAT3), patatin-like phospholipase domain-containing 3 (PNPLA3), peroxisome proliferator-activated receptor-gamma (PPAR), and peroxisome proliferator-activated receptor gamma coactivator 1 alpha (PPARGC1A), are linked to NAFLD development and progression^[33]^. Studies have shown that weakened rest-activity rhythms are associated with elevated liver enzyme levels [AST, alkaline phosphatase (ALP), gamma-glutamyl transferase (GGT)] and decreased albumin levels^[94]^. Extended periods of night work increased the vulnerability of nurses to dyslipidemia and abnormal liver and kidney function. When the hepatic circadian clock is not functioning optimally, the body becomes more susceptible to metabolic disruptions such as insulin resistance, heightened adiposity, and the progression of fatty liver diseases, diabetes, and obesity, as well as fibrosis and HCC^[39,90,95]^.

### Abnormal bile acid synthesis

Bile acid synthesis plays a key role as a signaling molecule in maintaining glucose, lipid, and energy balance, mediated by the farnesoid X receptor (FXR). This synthesis follows a daily rhythm governed by the circadian control of cholesterol 7α-hydroxylase expression and activity^[96]^. Chronic circadian disruption may cause the dysregulation of bile acids, resulting in altered nutrient utilization and storage, consequently promoting NAFLD development^[88]^. Rotating shifts perturb melatonin secretion, further disrupting circadian rhythms and potentially impacting liver metabolic homeostasis. This imbalance may lead to impaired nutrient utilization and increased accumulation through bile acid dysregulation. Kim *et al*. (2022) suggested a potential mechanism while studying Korean male steelworkers wherein reduced melatonin function against oxidative stress could contribute to the pathogenesis of NAFLD^[97]^.

### Altered glucose metabolism and insulin signaling in the liver

The liver maintains glucose balance, with its regulatory centers influenced by circadian rhythms that enable it to anticipate and effectively respond to changes in glucose levels during feeding and fasting^[98]^. During the active phase (daytime for humans and nighttime for mice), after a meal, the liver responds to high glucose levels by using glucose for glycolysis, or storing it as glycogen. However, upon reduced energy demands during the resting or fasting phase, the liver adapts to reduced energy demands by enhancing gluconeogenesis and glycogenolysis^[99]^. The disturbances to its circadian clock could have a significant impact on glucose regulation. Crucial regulators of these processes include glycogen synthase (GYS), glucose transporter 2 (GLUT2), glycogen synthase kinase (GSK3), and the insulin receptor (InsR)^[100]^. CRY, CGs, also modulate hepatic gluconeogenesis by influencing G protein-coupled receptor-mediated cyclic adenosine monophosphate (cAMP) accumulation and activating cAMP response element-binding protein (CREB)^[101,102]^. Circadian rhythm disruption may hinder insulin-induced Akt phosphorylation in white adipose tissue and affect the daily regulation of insulin sensitivity^[103]^. Importantly, disturbances in circadian rhythm are a leading cause of abnormal liver enzymes and insulin resistance among shift workers^[104]^.

### Altered lipid signaling in the liver

Approximately 20% of lipids, along with crucial enzymes involved in glucose, lipid, and bile acid metabolism, exhibit circadian oscillations in the mouse liver^[58]^. Hepatic CGs play roles in several key functions: (1) regulation of fatty acid (FA) synthesis via expression of elongation of very long chain FAs-3 (ELOVL3), long-chain FA family member 6 (ELOVL6), and Fas Cell Surface Death Receptor (Fas)^[105]^; (2) they influence beta-oxidation and ketone body production^[106]^; and (3) direct the expression of the vital lipid response NRs and peroxisome proliferator-activated receptors (PPARs)^[101,105,107]^. The involvement of the circadian machinery in lipid metabolism has resulted in its association with hepatic steatosis^[90]^. Mice exposed to simulated shift work develop hepatic steatosis, showing an increase in hepatic genes associated with FA and triglyceride (TG) synthesis, and a decrease in genes involved in beta-oxidation^[108,109]^. Limiting food intake during the circadian day in mice helps to reduce hepatic TGs via transcriptional and enzymatic regulation of FA synthesis, lipolysis, and β-oxidation by the clock genes Rev-Erbα, Per2, and PPAR-γ^[58,110]^. Furthermore, time-restricted feeding (TRF) protects against steatohepatitis by reducing proinflammatory long-chain FA production and increasing the synthesis of the antioxidant glutathione in the liver^[110]^. In contrast to the general population, nurses encounter heightened work pressure and lead significantly different lifestyles, including frequent night shifts and nocturnal meals, which constitute high-risk factors influencing NAFLD^[111]^.

## Factors That Initiate Nafld And Disease Severity In Night Shift Workers

Recent evidence strongly links unhealthy lifestyles and metabolic conditions such as exposure to LAN, rotating night shifts, sleep deprivation, smoking, lack of physical activity, erratic eating patterns, overweight, obesity, diabetes, and dyslipidemia to an increased likelihood of developing NAFLD^[111]^. Studies have consistently shown that longer durations and frequent NSW, especially among nursing staff, correlate with higher risks of increased weight and obesity^[112–115]^. The increased susceptibility to NAFLD among night-shift workers appears to be partly mediated by elevated BMI^[116]^. A cross-sectional study on Chinese steelworkers showed that a significant interaction exists between gender and the duration of night shifts in influencing NAFLD risk, with males exposed to longer shifts exhibiting particularly increased odds of developing the condition compared to their female counterparts^[52]^. Given these findings, greater emphasis should be placed on screening male night-shift workers for NAFLD, owing to their increased vulnerability to the disease.

Furthermore, a study involving over 12,000 Japanese individuals found that those who reported sleeping less than 5 h per night had a significantly higher risk of developing NAFLD compared to those who slept more than 7 h^[117]^. Insights from rodent models of shift work have shed light on the role of the peripheral clock in regulating liver function. Mice fed a high-fat diet and exposed to a rotating light schedule, mimicking shift work conditions, showed increased body weight and higher food intake during their inactive period compared to mice under a standard light schedule^[118]^. Moreover, after just 8 weeks of light schedule rotation, even on a standard chow diet, mice exhibited weight gain and hepatic steatosis, which worsened when they were subsequently fed a high-fat diet, leading to elevated levels of ALT^[119]^. Altered adipose tissue profiles marked by increased inflammation, angiogenesis, fibrosis, and adipocyte hypertrophy were seen in the adipose transcriptome of mice exposed to six months of rotating light schedules^[103]^. Continuous light exposure, mimicking circadian disruption by light pollution, exacerbated inflammation and insulin resistance induced by a high fructose diet in rats^[120]^. Nurses, particularly those in high-intensity and night-shift roles such as emergency departments, exhibit a notably high prevalence of NAFLD, with rates potentially reaching 28.3%^[121]^. To address the drawbacks associated with NAFLD, it has been proposed to reclassify the term NAFLD to metabolic dysfunction-associated fatty liver disease (MAFLD)^[122]^. The presence of hepatic steatosis in combination with at least one of the following three conditions - type 2 diabetes mellitus (T2DM), obesity, or metabolic dysregulation - are required for the diagnosis of MAFLD. Substantial evidence highlights that MAFLD is an independent risk factor for atherosclerosis and cardiovascular diseases. It is more closely associated with the primary risk factors for atherosclerosis and cardiovascular diseases, including dyslipidemia, T2DM, and hypertension, than NAFLD^[123]^. The new definition of MAFLD has emerged as metabolic dysfunction-associated steatotic liver disease (MASLD). While the exact mechanisms behind MASLD are not completely understood, disruptions in liver energy metabolism, lipid buildup, inflammation, and oxidative stress are believed to be the primary contributing factors^[124]^. Apart from these factors, recent preclinical studies have shown that the pertinent metabolic processes are regulated by circadian rhythms, underscoring the significant impact of circadian disruptions on the development of MASLD and metabolic dysfunction-associated steatohepatitis (MASH)^[125]^.

## Circadian Disruption Due To Night Shift: Nafld Initiation And Its Progression Into Nash

NSW leads to a misalignment between workers’ activity patterns and the liver’s natural circadian rhythm. This disruption may result in additional hepatocyte damage and subsequent liver function abnormalities^[101]^. This work-induced disruption could also worsen liver conditions such as fatty liver, cholestasis, hepatitis, cirrhosis, and hepatic cancer, while these conditions themselves can further disturb the body’s natural circadian rhythms^[126]^. The liver’s peripheral clock, regulated by the central clock, maintains its own independent rhythm, which is essential for liver homeostasis^[101]^. Scientific evidence shows that serum liver enzymes reach their lowest levels during the nighttime and peak during the daytime, indicating that the production of these enzymes follows a circadian rhythm^[127]^. Persistent disturbance of the circadian rhythm fundamentally alters the expression of thousands of genes that are differentially regulated in NASH and HCC. The transcriptomes initiate comprehensive activation of cancer hallmarks, leading to rapid progression from NAFLD to the development of HCC and subsequent metastasis, as observed in mouse model^[128]^. Moreover, NAFLD is linked to specific alterations in liver transcription factors, such as sterol regulatory element-binding protein (SREBP), which regulates lipid production, carbohydrate-responsive element-binding protein (ChREBP), involved in glucose balance maintenance; and nuclear factor kappa B (NF-κb), associated with inflammatory responses^[129]^. Scientists proposed a “two-hit hypothesis” to explain how NSW may cause liver damage in cases of NAFLD^[85]^. Initially, working night shifts triggers lipid accumulation in liver cells, a condition known as hepatic steatosis or non-alcoholic fatty liver (NAFL), making these cells more vulnerable to damage (“first hit”). Subsequently, the oxidative stress caused by NSW leads to liver cell death, which can progress to NASH and/or liver fibrosis (“second hit”)^[130]^. The “2-hit hypothesis” suggests that NAFL acts as a crucial intermediary in these mechanisms. The cancer-promoting effects of circadian disruption in NASH encompass metabolic reprogramming, persistent cell proliferation and inflammation, evasion of growth suppressors, DNA surveillance, and apoptosis, as well as enhanced transdifferentiation of hepatocytes and extracellular matrix dysregulation. Chronic disruption of circadian rhythms accelerates the progression of NASH to cirrhosis and fosters the development of precancerous environments. These environments are marked by inflammation that promotes tumor growth, increased formation of blood vessels, and activation of stroma-related epithelial-mesenchymal transition (EMT)^[39,131]^. The transcriptome analysis indicated that jet lag disrupts the regulation of oestrogen, androgen, WNT/β-catenin, transforming growth factor beta (TGFβ), phosphoinositide 3-kinase-Ak strain transforming-mammalian target of rapamycin (PI3K-AKT-mTOR), interferon, hedgehog, Interleukin-6-janus kinase 2-signal transducer and activator of transcription 3 (IL6-JAK2-STAT3), Notch, Interleukin-2-signal transducer and activator of transcription 5 (IL2-STAT5), and tumor necrosis factor-alpha-nuclear factor-kappa B (TNFα-NF-κB) signaling pathways in the liver, thereby contributing to the development of hepatic cancer^[128]^. Chronic exposure to disruptions in circadian rhythms can alter the expression of clock genes in the liver, potentially leading to various negative health effects, as reported in mice models^[132,133]^.

### Human population-based studies linking NSW to the initiation of NAFLD and NASH

NSW has been linked to deteriorated liver function and an increased risk of liver disease. Persistent rotating shift work frequently exhibits abnormal liver function, often indicated by irregular liver enzyme levels^[85]^. Yeung *et al*. found that disrupted rest-activity rhythms were linked to abnormal liver function biomarkers. Weakened rest-activity rhythms are correlated with poorer liver function, as indicated by various biomarkers, suggesting a potential role of circadian rhythms in maintaining liver health, as shown by the US National Health and Nutrition Examination Survey^[94]^. Furthermore, a 4-year longitudinal cohort study found a link between shift work and the incidence of NAFLD among the Chinese rail population, which supports the detrimental impact of shift work on NAFLD occurrence among these workers, especially those with frequent shift changes. The risk of developing NAFLD due to shift work was found to be higher in females and older individuals^[134]^. Shift work is associated with abnormalities in liver enzymes and provides some support for the notion that it has a greater impact on abnormal ALT levels in female workers compared to male workers in a cross-sectional study based on the Korea National Health and Examination Survey (2007-2015)^[135]^. The research examined the association between shift work and NAFLD in male workers within Korea’s steel manufacturing industry, revealing a link between shift work and moderate to severe NAFLD in these individuals^[97]^. Research involving male subjects indicated that individuals working night shifts had a higher likelihood of elevated ALT levels compared to those working during the day. Notably, a more pronounced dose-response effect was observed in individuals with longer durations of night shift employment^[40,41]^. NSW has been associated with the occurrence of NAFLD among Chinese female nurses, as evidenced by a web-based ambispective cohort study^[121]^. Similarly, rotating night shifts have been associated with a higher risk of new-onset NAFLD in Chinese male steelworkers. The study investigated how severe NAFLD correlates with prolonged QTc intervals and left ventricular hypertrophy (LVH) in a large group of Chinese male steelworkers. Significant correlations were found between the NAFLD fibrosis score, QTc interval abnormalities, left ventricular mass index (LVMI), and the likelihood of developing cardiovascular disease^[136]^. In line with this, blue-collar workers were reported to exhibit a higher incidence of prolonged QTc intervals, possibly attributable to factors such as overweight, obesity, smoking, and hypertension^[137]^. Lecca *et al*. discovered that consistent physical activity had a positive impact in preventing QTc prolongation^[138]^. Research on 6,881 steel production workers in China indicated that working night shifts was linked to higher chances of developing NAFLD, as revealed by a cross-sectional study. Specifically, current NSW was found to increase the risk of ultrasound-diagnosed NAFLD by 1.23 times^[52]^. Furthermore, two other Chinese cross-sectional studies have identified a link between rotating NSW and the occurrence of NAFLD, potentially attributed to disturbances in circadian rhythms^[45,52]^. In a Chinese occupational cohort, working night shifts that involve rotation was linked to an increased probability of NAFLD, as determined by ultrasound. This risk escalates with both prolonged periods of shift work and extended nighttime hours^[37]^. Working rotating night shifts is linked to higher levels of ALT, GGT, and other liver enzymes among male steelworkers^[139]^. However, shift work was not associated with NAFLD as determined by levels of liver enzymes, ALT and e-AST, in the two cross-sectional studies^[40,135]^. A Thai study examined the frequencies and duration of shift work and their relationship with NAFLD outcomes. While shift work status itself did not correlate with liver abnormalities, the length of time spent performing shift work was linked to NAFLD among workers^[140]^. A research study examined the relationship between different work schedules, individual biological rhythms, and their connection to liver fat accumulation and NAFLD among 282,303 participants from the UK Biobank. The study revealed that the hepatic proton density fat fraction (PDFF) was higher among irregular shift workers compared to those on permanent night shifts. Moreover, irregular shift work and a very late biological clock (chronotype) are linked to abnormal liver fat buildup, indicating that circadian disruption might play a role in its pathogenesis. These observations suggest implications for health strategies aimed at reducing the negative impact of shift work^[141]^. The research found NAFLD among healthy individuals working rotating shifts and explored its potential effects on nerve function. The findings suggest a significant prevalence of NAFLD among those engaged in rotating shift schedules. Furthermore, the study indicates that even though NAFLD is present, there appears to be no noticeable impact on the conduction of motor and sensory peripheral nerves, specifically in the common peroneal, median, and sural nerves^[142]^. Chinese research suggests that steelworkers who work shifts, particularly those with rotating schedules, are more prone to developing NAFLD due to disturbances in their internal body clock, which can contribute to liver fat accumulation and inflammation^[52]^. Interestingly, abnormal liver function, particularly indicated by elevated serum levels of ALT (e-ALT), is common among various professions that require NSW^[85,86]^. Workers on a 12-h rotating night shift showed notably elevated ALP levels and an increased risk of experiencing high to severe ALP levels compared to those on a fixed 12-h day shift^[41,143,144]^. After 5 years of monitoring Taiwanese electronic manufacturing workers, it was reported that 14.4% developed e-ALT. Additionally, those with hepatic steatosis initially showed that shift work could potentially worsen liver damage^[85]^. These investigations demonstrated a link between NSW and ALT levels, given the relevance of NAFLD to ALT levels^[85,86]^. Despite some uncertainty, research suggests a positive correlation between NSW and elevated ALT levels^[40,145]^. Similarly, serum ALP levels were elevated among shift workers, even after accounting for age, BMI, fasting blood sugar, and cholesterol levels^[144]^. The research discovered that both continuous exposure to light and a rotating light schedule triggered elevated levels of TNF-α, IL-6, IL-1β, along with serum lipids, AST, and ALT in mice^[146]^. Working night shifts by way of policemen specifically affects liver and kidney health, leading to conditions like NAFLD and declining renal function^[41,147]^. Biochemical markers that pertain to liver and kidney function primarily encompass ALT, AST, creatinine, uric acid (UA), and blood urea nitrogen (BUN).

### Animal-based study reflecting circadian clock disruption and risk of HCC

Rodents can shed light on the effect of jet lag and night shifts on CGs. The key circadian oscillators in the liver and skin of mammals were studied concerning the effects of short- or long-term exposure to rotating shift and chronic jet lag. Long-term rotating shift conditions cause a considerable alteration in the liver’s circadian clock in mice, with a less pronounced but still noticeable effect in mice experiencing chronic jet lag. On the other hand, the skin’s circadian clock is impacted by all three of the simulated shift environments, suggesting that the skin clock is more responsive to rotating shift-work light conditions over an extended period of time than the liver clock. According to the study’s findings, under conditions that mimic shift work, the skin’s canonical clock genes exhibit more profound disruptions than those of the liver. These findings imply that the skin clock is more susceptible to shift work’s negative consequences^[148]^. However, other studies showed how the circulating glucocorticoids rhythms are dissociated from lighting cues, causing disruption in the timing of feeding behavior in mice; this phenomenon is most typically reflected in shift work and “jetlag” in transmeridian travel. The findings demonstrate the unfavorable behavioral effects that can occur when two circadian systems receive anti-phasic cues; in this instance, the effect is on the control of glucocorticoids, which is a function that is as essential to health as feeding behavior^[149]^. Mouse-based models of human-like livers offer a robust preclinical platform to investigate how an inflamed liver environment and disrupted biological clocks contribute to hepatic cancer development, as well as to evaluate therapies against HCC^[128]^. The liver’s metabolism’s circadian homeostasis prevents carcinogenesis. Persistent disruption of circadian rhythms, like chronic jet lag, triggers spontaneous HCC in genetically normal mice, mirroring mechanisms seen in obese humans. Circadian disruption induces the activation of the constitutive androstane receptor (CAR) through mechanisms that promote cholestasis, peripheral clock disruption, and sympathetic dysfunction. Consequently, circadian dysfunction emerges as a significant risk factor for HCC. The research demonstrates that long-term disruption of biological rhythms alone can lead mice to develop hepatic cancer spontaneously, without changes in diet, external genetic stressors, or inherited genetic mutations^[39]^. Michelotti *et al*. reviewed studies and demonstrated that jet lag in mice leads to a widespread alteration in liver metabolism. This alteration not only enhances the production and storage of fats through increased cytoplasmic glycolysis but also elevates oxidative stress and boosts the synthesis of biochemical building blocks that facilitate fast cell division. Most of the jet-lagged wild-type mice exhibit the advancement from NAFLD to NASH and subsequently to fibrosis at a young age. This is probably because jet lag induces sustained liver damage and inflammation, initiating a prolonged regenerative wound-healing cycle, which is a typical mechanism for tumor initiation^[150]^.

## Prolonged Working Hours And Extended Years Of Shift Work: Progression Of Nafld/Nash Into Disease Severity

A cross-sectional study in southern China found that extended rotating NSW is associated with an increased risk of dyslipidemia and abnormal liver function in nurses^[151]^. Other studies also reported cardiovascular disease^[152,153]^, diabetes mellitus^[154]^, MetS^[155]^, and obesity in long-working shift workers^[156]^. In recent studies, including the Korea National Health and Nutritional Examination Survey VII and a large population-based Korean study, long work hours have been linked to NAFLD and recognized as an independent risk factor^[42,52,157,158]^. It is speculated that overwork contributes to the pathophysiology of NAFLD. Long working hours are strongly associated with psychosocial stress in blue-collar workers^[159]^. Moreover, a study revealed that the lean NAFLD group exhibited a higher incidence of working more than 40 h per week yet sleeping for shorter periods of time compared to the control group^[160]^. Notably, extended working hours were strongly correlated with a higher incidence of lean NAFLD in the Korean population with long working hours and its severe manifestations in men, whereas this association was not observed in women^[161]^. Indeed, workers who occasionally or consistently worked night shifts had a 1.12 and 1.27 times higher likelihood, respectively, of developing NAFLD compared to those who never or rarely worked night shifts. Additionally, prolonged work hours, particularly those exceeding 60 h per week, were independently linked to the onset of NAFLD^[158]^. In a prospective study, the analysis of 281,280 UK Biobank participants showed that extended periods, increased frequency, consecutive night shifts, and prolonged hours per shift were linked to elevated risks of NAFLD, with risk escalating as these factors intensified. The study determined that the correlation between NSW and the occurrence of NAFLD remained significant regardless of genetic predisposition to the disease^[162]^. More intriguingly, the Dallas Steatosis Index (DSI) has been used in prior research to estimate NAFLD prevalence in biobank studies, such as those conducted with the UK Biobank^[163]^. Apart from this, a cross-sectional study utilizing data from the Korea National Health and Nutrition Examination Survey VII, which included 5,661 employed adults with no history of liver disease or heavy alcohol consumption, demonstrated that longer working hours are associated with an increased prevalence of NAFLD as indicated by a Hepatic Steatosis Index (HSI) score of 36 or higher. Individuals who worked 53-83 h per week had a greater likelihood of developing NAFLD compared to those who worked the standard 36-42 h per week, even after accounting for variables such as age, sex, BMI, smoking, alcohol consumption, physical activity, diabetes mellitus, hypertension, serum TG levels, and total cholesterol. Scientific studies have shown that increased physical inactivity^[164]^ and reduced leisure-time physical activity, both of which are exacerbated by long working hours, are linked to lean NAFLD, as reported in a cross-sectional study of South Korea^[165]^. Additionally, irregular shift work has been associated with the accumulation of pathological liver fat (hepatic fat fraction) and NAFLD in a study with 282,303 UK Biobank participants^[141]^ and disease severity.

## Carcinogenic Effect Of Night Shift On Hcc And Other Malignancies

Long-term disruptions in circadian rhythms increase the likelihood of HCC associated with NAFLD, although the precise mechanisms and direct implications for human HCC remain unclear. HCC, previously considered uncommon in the United States and other developed nations, has seen a nearly threefold rise in incidence, accompanied by a faster increase in cancer-related deaths^[166]^. In the context of liver metabolic disorders, NAFLD is projected to become the primary cause of HCC in the 21st century due to the widespread obesity epidemic^[167]^. Circadian disruption is associated with a higher cancer risk due to effects on the circadian system, including disturbances to its functioning and the activation of oncogenes, while also suppressing tumor suppressor genes. Using surveillance data, studies examined the relationship between age-standardized country-level cancer incidence rates for each cancer combined and 23 specific cancers by gender and the location inside a given time zone. The findings suggest that the timing of a place’s location concerning time zones provides a fascinating angle on how long-term disruptions to circadian rhythms may raise the risk of cancer. It appears that living near the western edge of a time zone in the United States significantly raises the risk of HCC, even after controlling for lifestyle factors like obesity^[38,168]^. Persistent disruption of daily biological rhythms can independently promote hepatic cancer in human liver cells. Liang *et al*. presented interesting observations and systematically analyzed the possible functions of 13 core circadian clock genes (CCGs) in HCC to identify ideal biomarkers and therapeutic targets. Patients with low levels of CRY2, PER1, RORα, or high expressions of TIMELESS have a bad prognosis. Furthermore, pathway analysis showed that FA metabolism, the PI3K/AKT pathway, and the cell cycle are all impacted by these four CCGs. According to this research, the circadian clock genes TIMELESS, CRY2, PER1, and RORα may be used as therapeutic targets, diagnostic and prognostic biomarkers, and indicators of prognosis for patients with HCC. These genes may also facilitate HCC chronotherapy by rhythmically controlling drug sensitivity and crucial cellular signaling pathways. Overall, this disease progression closely resembles the pathophysiological process seen in humans with NAFLD that leads to spontaneous hepatic cancer^[169]^. One significant alteration in modern society’s way of life resulting from globalization and industrialization is the prevalence of chronic social jet lag and NSW^[170,171]^. Rotating shift work could potentially elevate the susceptibility to prostate cancer^[172]^, breast cancer^[173–175]^, lung^[176,177]^, bladder cancer^[178]^, biliary tract cancer^[179]^, or colorectal cancers^[180,181]^.

### Circadian rhythms-mediated dysfunction in liver metabolism

Circadian rhythms of our body’s physiological functions are synchronizing with external environmental rhythms. These rhythms influence a variety of hepatic functions, including liver metabolism and homeostasis. Disruption in the circadian rhythm of physiological functions at the cellular or tissue level can lead to a number of diseases associated with hepatic health and metabolism. Tables 1 and 2 provide research studies conducted on circadian rhythm-mediated dysfunction in liver metabolism.

## Strategies To Reduce The Risk Of Nafld/Nash Progression And Disease Severity In Shift Workers

In the realm of metabolism, most attention has been directed toward nutrition and exercise, while controllable hazards from circadian disruption have received relatively little focus. Enhancing sleep quality by ensuring adequate daytime sleep duration is crucial for night shift workers. It would be advisable to recommend that night shift workers with NAFLD aim for 7 to 9 h of daytime sleep as part of their healthy lifestyle modifications. Furthermore, research has shown that irregular snacking after midnight - a common eating pattern among Japanese night shift workers - is associated with elevated levels of 8-isoprostane^[182]^. Night shift workers should refrain from snacking at midnight. Embracing a healthier lifestyle, including increased physical activity and improved nutrition, can help lower the risk of metabolic disorders in these workers^[183]^. However, its effectiveness in reducing cancer risk has yet to be proven. The primary strategy for treating NAFLD centers on altering lifestyle behaviors. This includes encouraging adherence to the Mediterranean diet (MedDiet), which is abundant in carotenoids, polyphenols, fiber, and polyunsaturated FAs, while avoiding refined and high-sugar foods. In addition, regular physical activity is recommended to promote weight loss and manage the cardiometabolic risk factors linked to MetS effectively^[184,185]^. Night shift workers could be encouraged to eat either at the beginning or toward the end of their shift while avoiding meals during the biological night (e.g., midnight to 6:00 a.m.). They should also refrain from consuming large meals just before their daytime sleep^[186]^. On the other hand, effective strategies to enhance both the length and quality of sleep involve utilizing subdued lighting, taking brief naps, reducing exposure to electronic screens, and minimizing consumption of stimulants like caffeine, alcohol, tobacco, and snacks while working night shifts. Besides this, substantial evidence highlights that exposure to light during the nighttime and disturbances in circadian rhythms decrease the production of melatonin in individuals working night shifts. As a proof of concept, melatonin possesses oncostatic properties by acting as an antioxidant, stimulating apoptosis, scavenging free radicals, and inhibiting angiogenesis^[187,188]^. Thus, it could potentially serve as adjuvant therapy for various cancers^[189]^. According to two recent publications on rodent models, melatonin therapy decreases liver cell growth and oxidative stress while promoting programmed cell death in rats treated with diethylnitrosamine (DEN)^[190,191]^. The proposed perspective for the treatment of circadian rhythm-mediated dysfunction in liver metabolism includes lifestyle changes, melatonin supplementation, etc. A novel and efficient intervention emerged based on time of eating with circadian alignment termed TRF. This behavioral intervention can synchronize the central and peripheral rhythms, which in turn can treat circadian metabolic rhythm dysfunction and hepatic steatosis. The hormone melatonin supplementation can improve NASH and can also help regulate cholangiocyte functions and maintain liver homeostasis.

Some recent research findings progressively showed the prevention and treatment of circadian rhythm-mediated dysfunction in liver metabolism. Circadian proteins regulate several other proteins that are presently being investigated as potential therapeutic targets in NAFLD, including acetyl-CoA carboxylase (ACC), PPARs, incretins, and SREBP. As a result, the clock can maximize the positive effects and minimize the adverse effects of pharmacological medicines. The clock itself has the potential to treat circadian alignment as a target for NAFLD^[90]^. Since the oscillator responds to reset or resynchronize stimuli and certain clock components are directly involved in metabolic processes, small compounds targeted at clock components may offer other targets for therapeutic intervention^[192,193]^. Autophagy targets CRY1 for degradation, which has the temporal effect of suppressing hepatic gluconeogenesis^[194]^. The circadian clock’s functional network extends to regulate numerous physiological processes, including those targeted by almost all medications for MetS spectrum illnesses^[195]^. One possible target for the therapy of NAFLD is the circadian clock’s rhythmic influence on drug metabolism and detoxification processes through chronomedicine or chronopharmacokinetics^[33,126]^.

## Concluding Perspective

Circadian rhythms are based on a conserved, self-sustaining molecular clock present in all mammals, including humans. This clock exists in nearly all tissues and cells, underscoring the importance of temporal organization at both the cellular and organismal levels. Occupations requiring permanent NSW often involve inappropriate eating times, exposure to LAN from artificial lighting or blue light-emitting devices, and frequent consumption of arousal-stimulating beverages like coffee and snacks during the night. These factors are well-established disruptors of the circadian system, progressively altering daily life. Misalignment in behavioral and physiological rhythms due to long hours or longer years of NSW is increasingly recognized as the accumulation of hepatic fat, abnormal liver function, progression and pathology of inflammation-related diseases, predominantly NAFLD/MASLD, and the risk of hepatic cancer.

Potential breakthroughs in this area will enhance scientific knowledge and improve occupational health. In this context, public and occupational health policies aim to raise awareness of the risks associated with unscheduled lifestyle-related diseases and the management of biological rhythm disorders. These policies will also guide employers in fostering a work environment that allows employees to perform at their best without compromising their health. Further research could focus on targeting genes such as CRY, Per, Rev-Erb, and ROR alpha through loss-and-gain experiments (e.g., using Rev-Erb alpha agonists), which may lead to novel drug targets for treating insulin resistance and hepatic metabolism. Such drugs could be beneficial in the treatment of NAFLD and NASH.

## Figures and Tables

**Table 1:** Animal studies showing circadian rhythm-mediated dysfunction in liver metabolism

S. No.	Study model system/population	Observation	Outcome	Reference
1	Mouse model	Experimental chronic jet lag.	Persistent dysregulation of liver metabolism. Cholestasis, peripheral clock disruption, and sympathetic dysfunction. Spontaneous HCC development.	[39]
2.	Rat model	Shift in food intake during simulated night-shift work on liver metabolism.	Development of hepatic steatosis. Increase in FA and TG synthesis. Decrease in beta-oxidation.	[109]
3.	Mouse model	Circadian clock, feeding time and lipid homeostasis.	Circadian clocks and feeding time dictate the phase and levels of hepatic TG accumulation.	[58]
4.	Mouse model	Mechanisms of sleep deprivation-induced glucose intolerance on liver function.	Increased hepatic glucose production. Increased lipid oxidation. Hepatic steatosis and insulin resistance.	[76]
5.	Mouse model	Modeling shift work via a rotating light cycle.	Promotes weight gain. Increased hepatic glycogen and TG.	[119]
6.	Rat model	Constant light exposure to normal chow or high-fat diet-fed animals.	Glucose abnormalities and dyslipidemia in normal chow-fed rats. Glucose abnormalities, dyslipidemia, insulin resistance, inflammation and aggravated steatohepatitis in high fat fed rats.	[120]
7.	Mouse with humanized livers	Circadian dysregulation via chronic jet lag.	Circadian dysfunction induces glucose intolerance, NAFLD-associated human HCCs, and human HCC metastasis independent of diet in a humanized mouse model.	[128]
8.	Mouse model	Chronic circadian disruption via environmental uncoupling of the light-dark phases.	Created pathology similar to the genetic circadian disruption observed with loss of SRC-2. Metabolic syndrome and NAFLD.	[132]

HCC: Hepatocellular carcinoma; FA: fatty acid; TG: triglyceride; NAFLD: non-alcoholic fatty liver disease; SRC: steroid receptor coactivator.

**Table 2:** Human epidemiological studies showing circadian rhythms-mediated dysfunction in liver metabolism

S. No.	Study model system/population	Observation	Outcome	Reference
1.	Human (US-based population)	Association of rest-activity rhythms and liver function.	Elevated ALP and GGT, lowered albumin.	[94]
2.	Human (Korean steelworkers)	Correlation between shift work and NAFLD	Shift workers have moderately severe NAFLD.	[97]
3.	Human (US-based population)	Time zone meridian and HCC risk	Circadian misalignment from the western region of a time zone impacts hepatocarcinogenesis.	[38]
4.	Human (night shift workers from South China)	Rotating night shift	High levels of ALP enzyme and abnormal liver function.	[41,144]
5.	Human (Korean population)	Long working hours	Pronounced risk of NAFLD.	[42]
6.	Human (Steelworkers of China)	Rotating night shift	Elevated ALT, GGT, and increased liver enzymes.	[52,139]
7.	Human (UK-based population)	Shift work evening/late chronotype	Hepatic fat accumulation and circadian misalignment.	[57,141]
8.	Human (Chinese population)	Nighttime sleep duration	Long nighttime sleep duration is associated with a modestly increased risk of NAFLD.	[75]
9.	Human (US adult population)	Sleep deprivation (duration and quality).	Optimal sleep duration (≥ 7 h) is associated with a lower likelihood of abnormal ALT levels and NAFLD.	[77,78]
10.	Human (Chinese male night shift workers)	NSW	NSW is associated with elevated ALT levels.	[87]
11.	Human (Japanese population-based study)	Effect of sleep duration	Short sleep duration is a significant risk factor for NAFLD.	[117,157]
12.	Human (Chinese rail workers)	Shift work	Harmful effect of shift work on NAFLD incidence.	[134]
13.	Human (Korean population)	Shift work	Shift work affects female workers more than males on abnormal ALT.	[135]
14.	Human (Thai Shift workers)	Duration of shift work	Liver enzyme abnormalities and NAFLD.	[140]
15.	Human (Korean population-based study)	Long working hours.	Long working hours are independently associated with incident NAFLD.	[158,161]

ALP: Alkaline phosphatase; NAFLD: non-alcoholic fatty liver disease; HCC: hepatocellular carcinoma; ALT: alanine aminotransferase; NSW: night shift work; GGT: gamma-glutamyl transferase.

## Data Availability

Not applicable.
